# Intravital imaging allows real-time characterization of tissue resident eosinophils

**DOI:** 10.1038/s42003-019-0425-3

**Published:** 2019-05-13

**Authors:** Andrew Chojnacki, Katarzyna Wojcik, Björn Petri, Gurpreet Aulakh, Elizabeth A. Jacobsen, William E. LeSuer, Pina Colarusso, Kamala D. Patel

**Affiliations:** 1 Department of Physiology and Pharmacology, Snyder Institute for Chronic Diseases, Cumming School of Medicine, University of Calgary, Calgary, AB Canada; 2 Department of Microbiology, Immunology and Infectious Diseases, Snyder Institute for Chronic Diseases, Cumming School of Medicine, University of Calgary, Calgary, AB Canada; 3 Western College of Veterinary Medicine, University of Saskatchewan, Saskatoon, SK Canada; 4 Division of Allergy and Clinical Immunology, Mayo Clinic Arizona, Scottsdale, AZ USA; 5 Department of Biochemistry and Molecular Biology, Snyder Institute for Chronic Diseases, Cumming School of Medicine, University of Calgary, Calgary, AB Canada

**Keywords:** Imaging the immune system, Chronic inflammation

## Abstract

Eosinophils are core components of the immune system, yet tools are lacking to directly observe eosinophils in action in vivo. To better understand the role of tissue resident eosinophils, we used eosinophil-specific CRE (eoCRE) mice to create GFP and tdTomato reporters. We then employed intravital microscopy to examine the dynamic behaviour of eosinophils in the healthy GI tract, mesentery, liver, lymph node, skin and lung. Given the role of eosinophils in allergic airway diseases, we also examined eosinophils in the lung following ovalbumin sensitization and challenge. We were able to monitor and quantify eosinophilic behaviours including patrolling, crawling, clustering, tissue distribution and interactions with other leukocytes. Thus, these reporter mice allow eosinophils to be examined in real-time in living animals, paving the way to further understanding the roles eosinophils play in both health and disease.

## Abstract (web summary)

Andrew Chojnacki et al develop reporters to enable real-time in vivo and ex vivo imaging of tissue-resident eosinophils inside several organs in mice. They describe eosinophil behaviours such as active patrolling of the lymph nodes, crawling and clustering.

## Introduction

Dogma holds that eosinophils cause damage to organs like the gut, skin, and lung by releasing toxic granular proteins that are otherwise used to defend against parasites. We now know that their roles are far more complex^1–6^. Over the past decade, we have learned that eosinophils have diverse roles depending on site of infiltration, time course of recruitment, and degree of activation^3,4,6–9^.

The complexity of eosinophil biology is illustrated by its influence on development, homeostasis and dysregulation within different organ systems during disease. For example, eosinophils are normally found throughout most of the gastrointestinal (GI) tract in both humans and mice^10–12^. Mice genetically deficient in eosinophils have altered Peyer’s patch development^13^, fewer lymphoid cells in the lamina propia^13^, make less secretory IgA^13,14^, and have altered mucosal barrier function^13^. These data suggest that eosinophils participate in key homeostatic functions in the GI tract. The data on eosinophils in models of inflammatory bowel diseases are conflicting, with some studies showing a protective role for eosinophils^15^, whereas others suggest they are destructive^16,17^.

In the lung where eosinophils have been heavily studied, recent data also challenge the concept of eosinophils acting exclusively as tissue-damaging effector cells. Eosinophils have a protective role following viral challenge to pneumovirus^18^ and parainfluenza^19^. In allergic airway models, eosinophils can migrate into the lung draining lymph nodes with kinetics similar to dendritic cells, and once there, they take up residence in the T cell-rich zones^20^, which may account for eosinophil-mediated effects on T cell responses during airway inflammation^4^. Eosinophils can also influence T cell polarization by releasing T helper type 2 (Th2) cytokines and suppressive mediators resulting in suppression of Th1 and Th17 responses^20^. Moreover, eosinophils influence tissue remodeling by releasing mediators like transforming growth factor β1^3^. Finally, a small population of lung-resident eosinophils exists, but their role in health and disease remains largely unknown^21^.

These recent advances in the study of eosinophil biology were only possible through the rapid expansion of eosinophil-focused antibodies, genetically modified mice and improved technologies. Despite this, uncertainty still surrounds the eosinophil. The current standard for identifying tissue-resident eosinophils in vivo involves either histology or flow cytometry (reviewed in ref. ^2^) and both are fraught with issues. Histology is notorious for underestimating tissue eosinophils in part due to inconsistent labeling by common stains that target granule contents. Using antibodies against major basic protein (MBP)^22^ or eosinophil peroxidase (EPX) has improved this situation, but we are still constrained by the use of thin sections and an inability to fully survey intact, living tissue. Flow cytometry has helped in characterizing tissue eosinophil number and distribution^23^, yet these studies are still limited because the tissue architecture is lost and eosinophils can be affected by tissue digestion^23^.

Intravital microscopy is a powerful tool for directly visualizing immune cells in real time^24,25^, yet experiments with eosinophils have been plagued by an inability to specifically label endogenous eosinophils. Some previous studies required removal and isolation of eosinophils from interleukin (IL)-5 transgenic mice^26^. These cells were then labeled exogenously and re-injected back into the animal^26^. Although helpful in understanding eosinophil recruitment in acute inflammation, examining the behavior of steady-state tissue-resident eosinophils or eosinophils during ongoing chronic inflammation was not possible.

To overcome this challenge, we took advantage of the eosinophil CRE (eoCRE) mouse developed by Doyle et al.^27^. These mice have CRE recombinase inserted into the *epx* gene^27^. CRE was expressed in virtually all eosinophils and expression was exquisitely specific^27^. Using the eoCRE mice, we generated eosinophil-specific fluorescent reporter animals and performed intravital microscopy^24^. We have now been able to directly observe, in real time, the distribution and behavior of tissue-resident eosinophils in healthy mice as well as those with allergic airway inflammation. Though a powerful tool for understanding dynamic behavior, intravital microscopy is limited in terms of field of view, tissue penetration and surgical accessibility, thus we complemented these studies with whole-organ fluorescent imaging. Together these innovative tools are giving us a new glimpse into the enigma that is the eosinophil.

## Results

### Eosinophils in the healthy GI tract

**Fig. 1:**
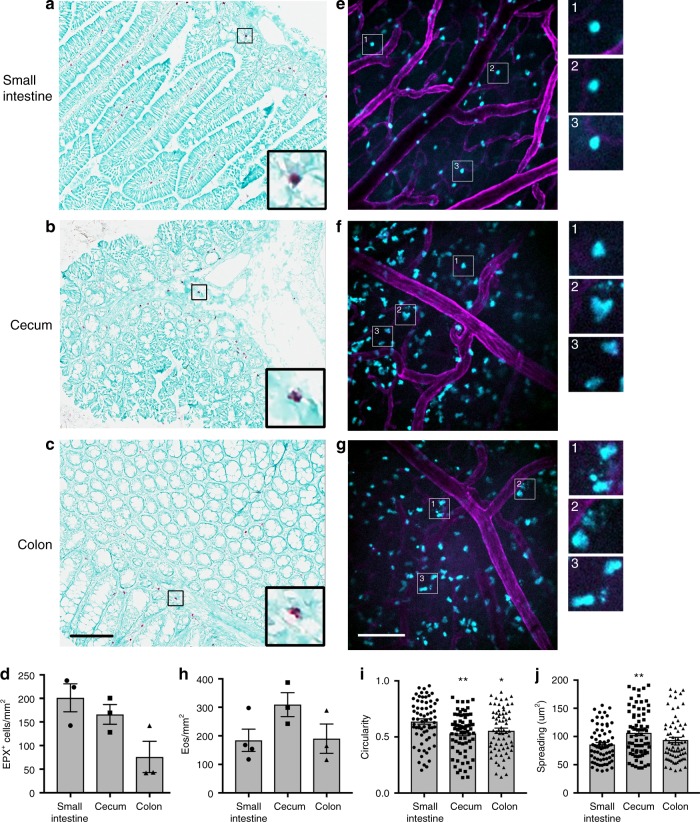
Eosinophils are expressed throughout the gastrointestinal system under baseline conditions. **a**–**d** Tissues from wild-type C57Bl/6 mice were isolated, fixed, sectioned and then stained with an anti-eosinophil peroxidase (anti-EPX) antibody as described in the “Methods.” Tissue slices were imaged on a digital pathology slide scanner using a ×20/0.75 NA objective. Representative images are shown in **a**–**c**. The number of EPX-positive cells was determined and the data shown in **d** are the mean ± SEM and individual values of *n* = 3 independent experiments. **e**–**h** eoCRE^+/−^/GFP^+/−^ mice were anesthetized and the specified organs were prepared for intravital microscopy as described in the “Methods.” Eosinophils express green fluorescent protein (cyan) and the vasculature is labeled with an anti-CD31 antibody conjugated to Alexa-594 (magenta). Representative images from the small intestine (**e**), cecum (**f**), and colon (**g**) were captured from video sequences. These data were then quantified and the results are shown in **h**. Images of individual eosinophils and/or structures consistent with intact free granules are shown in greater magnification in the insets to the right of each full frame. The circularity and spreading of the imaged eosinophils was determined and the data are shown in **i** and **j**. Data in **h** represent mean ± SEM; *n* = 3 independent experiments (Cecum, Colon) or n = 4 independent experiments (Small intestine). Data in **i** and **j** represent the mean ± SEM; *n* = 68 (Small intestine and Colon) and *n* = 73 (Cecum) cells measured. Data in **i** *p = 0.0217 and **p = 0.0021 as compared to the small intestine. Data in **j** **p = 0.0023. Scale bar for **a**–**c** is shown in **c** and is 100 μm. Scale bar for **e**–**g** is shown in **g** and is 100 μm

Eosinophils are resident in most of the GI system^10^. We confirmed this by performing immunostaining in the small intestine, cecum, and colon of wild-type animals using an anti-EPX antibody (Fig. 1a–c). These data show that eosinophils are present throughout the small and large intestines, with more eosinophils in the small intestine as compared to the colon (Fig. 1d). We then used spinning-disk confocal microscopy to examine eosinophils in the GI tract of living animals under baseline conditions in eoCRE^+/−^/GFP^+/−^ mice. The submucosal vessels of the small intestine, cecum and colon were visualized by labeling the vessels with an anti-CD31 antibody (magenta). We then examined the muscularis layer of the GI tissue for green fluorescent protein (GFP)-expressing eosinophils (cyan). A major challenge when performing fluorescent intravital microscopy is having target cells that are bright enough to allow for high-speed imaging (≥20 fps). Eosinophils expressing GFP were exceptionally bright when examined by flow cytometry ex vivo^27^ and this translated in vivo, enabling us to effectively visualize eosinophils in all of these compartments (Fig. 1e–g).

Quantifying the tissue-resident eosinophils showed that there was similar number of eosinophils per unit area in the small intestine, cecum, and colon (Fig. 1h). Despite similar numbers, the morphology of the eosinophils in the small intestine was different than those in the cecum or colon. Eosinophils in the small intestine had a rounded morphology (Fig. 1e with magnified inset images 1–3), whereas those in the cecum and colon were spread and often had numerous motile projections (Fig. 1f, g, with magnified inset images 1–3). Circularity and spreading were quantified and the difference in eosinophil morphology between these compartments of the GI tract was significant (Fig. 1i, j). We also observed small, punctate, fluorescent structures in the cecum, and to a greater extent, in the colon (Fig. 1f inset 2 and Fig. 1g insets 1–3). These small structures are consistent with intact eosinophil granules^3,22,28^.

### Eosinophils visualized in multiple organ systems

**Fig. 2:**
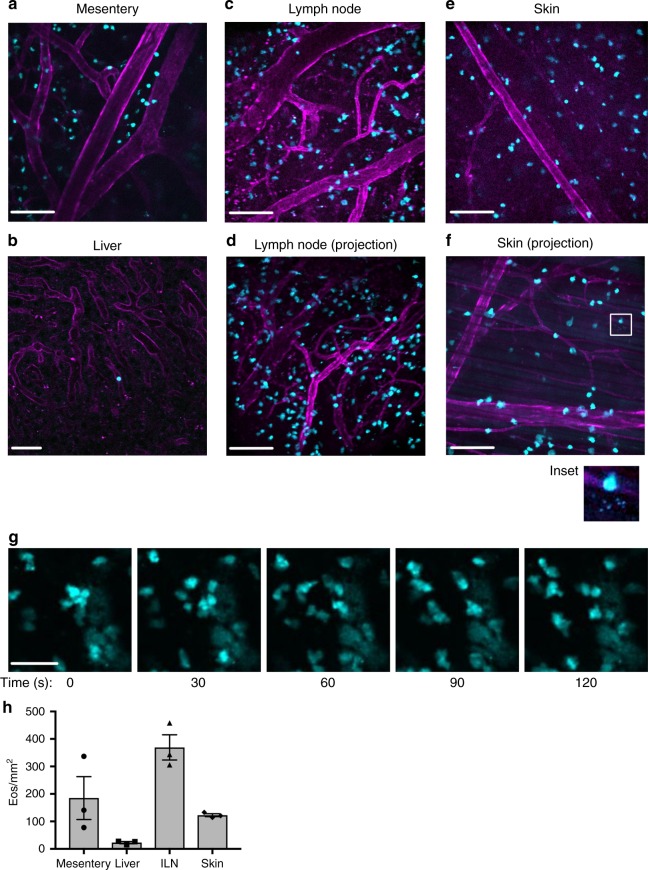
Tissue-resident eosinophils can be visualized in real time under baseline conditions in multiple organ systems using intravital microscopy. EoCRE^+/−^/GFP^+/−^ mice were anesthetized and the specified organs were prepared for intravital microscopy as described in the “Methods.” Eosinophils express green fluorescent protein (cyan) and the vasculature is labeled with an anti-CD31 antibody conjugated to Alexa-594 (magenta). Representative images from the mesentery (**a**), liver (**b**), inguinal lymph node (**c**, **d**), and skin (**e**, **f**) were captured from video sequences. **d** and **f** are maximum intensity projection images generated using the optical sections from the *z*-stack of the tissue. Inset in **f** shows small punctate structures consistent with free granules. **g** Eosinophils from the lymph node are shown over a 90-s time frame. The full 6-min video is shown as supplementary video 1. Images are representative of between three and five experiments. Data in **h** are mean ± SEM; *n* = 3 independent experiments. Scale bars in **a** and **c**–**f** are 100 μm, scale bar in **b** is 50 μm and scale bar in **g** is 40 μm

We next examined tissue eosinophils across multiple organ systems using intravital microscopy beginning with the mesentery. The mesentery connects the intestines to the abdominal wall and was recently described as its own contiguous organ^29,30^. To date, the presence of eosinophils in the mesentery has not been well defined. The mesenteric tissue was surgically exposed and examined for the presence of GFP^+^ eosinophils. We found robust numbers of eosinophils within the mesenteric tissue (Fig. 2a and quantified in Fig. 2h). We also examined the liver, lymph node, and skin. Only a few sporadic eosinophils were found in the liver (Fig. 2b, h). This was consistent with anti-EPX staining (Supplementary Fig. 1a, e). In contrast, the inguinal lymph node was rich in eosinophils under baseline conditions and a significant number of eosinophils were also found in the skin (Fig. 2c–f and quantified in Fig. 2h). This was observed in individual optical sections as well as in maximum intensity projection images of tissue that had been optically sectioned (lymph node: Fig. 2c, d; skin: Fig. 2e, f). Similar to the GI tract, we found evidence for cell-free granules in the skin. The maximum projection image in Fig. 2f in particular shows a group of these structures separate from any nearby eosinophils, suggesting that these small bright structures are not coming from adjacent cells or cells in a different focal plane (Fig. 2f, inset). High-resolution laser scanning confocal microscopy of blood eosinophils from eoCRE^+/−^/GFP^+/−^ mice showed that tdTomato was found throughout the cytoplasm and was not excluded from granules (Supplementary Fig. 2), suggesting that intact granules could be capable of fluorescing. Eosinophils were also observed patrolling under baseline conditions in many of the tissues examined, as demonstrated by time-lapse video. Figure 2g and supplementary video 1 specifically show eosinophils patrolling the lymph node.

Observing large numbers of eosinophils in the skin was a surprise, as eosinophils are generally not thought to be present in the skin under baseline conditions^31^. Data from Yu et al. supported this by showing that eosinophil only comprise 5–6% of leukocytes in the skin using flow cytometry^23^. Notably, Yu et al. used skin from the ears, whereas we examined eosinophils in a skin flap on the dorsal median side of the mouse^23^. To address these conflicting data, we made use of whole-body imaging to screen whole organs ex vivo. Whole-organ screening takes advantage of the fluorescent reporter mice, yet queries the tissue differently than intravital microscopy, providing more expansive organ coverage albeit at lower resolution.

**Fig. 3:**
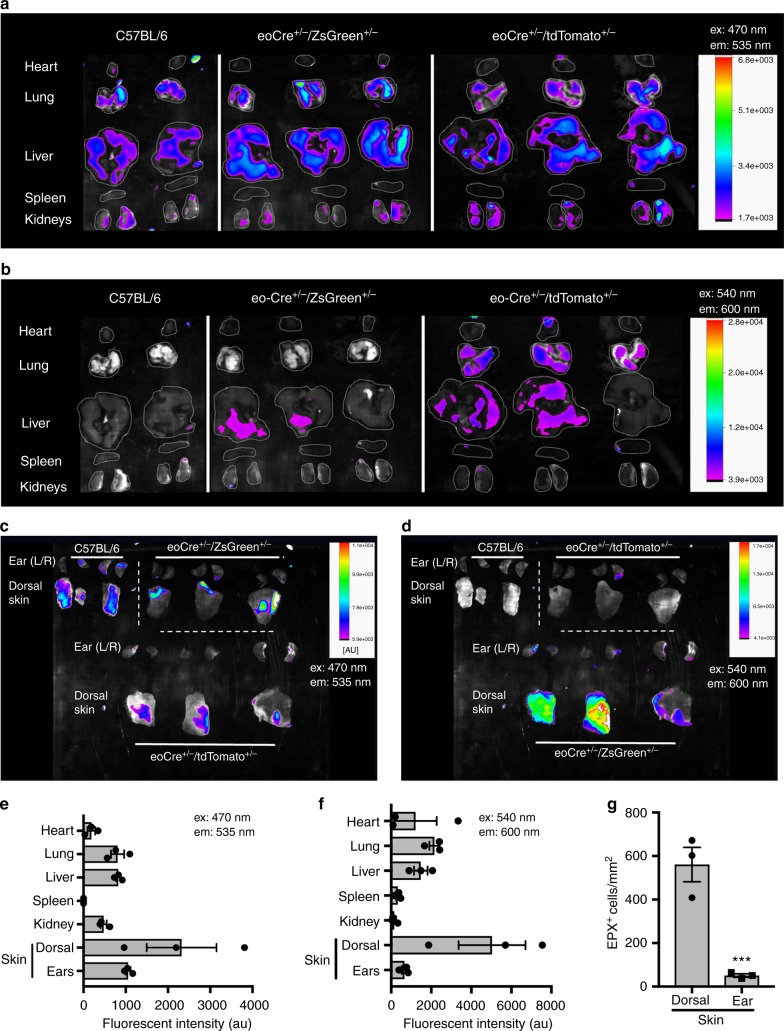
Whole-organ imaging of tissue-resident eosinophils. The organs from wild-type, eoCRE^+/−^/eGFP^+/−^, or eoCRE^+/−^/tdTomato^+/−^ mice were removed and directly imaged in the green channel (**a** and **c**, ex:470 nm, em: 535 nm) or in the red channel (**b** and **d**, ex: 540 nm, em: 600 nm) using an InVivo Xtreme imaging system as described in the “Methods.” Fluorescent images are shown in color, superimposed over a black and white image of the organs. **e**, **f** Fluorescence emission signal in the green (**e**) and red (**f**) channels was quantified and corrected for background. Data represent mean ± SEM, *n* = 3 animals. **g** Skin from the dorsal flap or the ears were stained for eosinophil peroxidase and the number of eosinophils was determined as described in Fig. 1. Data represent mean ± SEM and individual values, *n* = 3 animals. ***p < 0.001

The organs from wild-type C57Bl/6, eoCRE^+/−^/GFP^+/−^, or eoCRE^+/−^/tdTomato^+/−^ mice were dissected and then visualized in either the green emission channel (Fig. 3a, c, ex: 470 nm and em: 535 nm) or in the red emission channel (Fig. 3b, d, ex: 540 nm and em: 600 nm) using an InVivo Xtreme 4MP whole-body imaging system. Background fluorescence in wild-type mice was high in the green channel leading to a low signal-to-noise ratio (Fig. 3a, c). In contrast, background fluorescence in wild-type mice was quite low in the red channel resulting in an improved signal-to-noise ratio when eoCRE^+/−^/tdTomato^+/−^ mice were examined (Fig. 3b, d). These data were quantified and the resulting data in the green and red emission channels were qualitatively equivalent (Fig. 3e, f).

We found very low levels of fluorescence in the heart, kidney, or spleen even though these are highly vascular organs (Fig. 3a, b, e, f). There were low but measurable levels of fluorescence in the liver and lung (Fig. 3a, b, e, f). The skin and liver were examined using both intravital microscopy and whole-organ imaging and these methods showed a similar trend, with 3–5 times more eosinophils in the dorsal skin flap as compared to the liver (Figs. 2h and 3e, f). When we examined the ears, we found very little fluorescence, consistent with flow cytometric and histological data, yet when the dorsal skin flap was examined we found much higher levels of fluorescence (Fig. 3c, d). We found a similar difference in the number of eosinophils in the ears versus the dorsal region using EPX immunostaining in wild-type mice (Fig. 3g and Supplementary Fig. 1). These data suggest that, even within a particular organ, eosinophil numbers vary depending on the specific anatomical site.

### Intravital imaging of lung eosinophils in health and disease

**Fig. 4:**
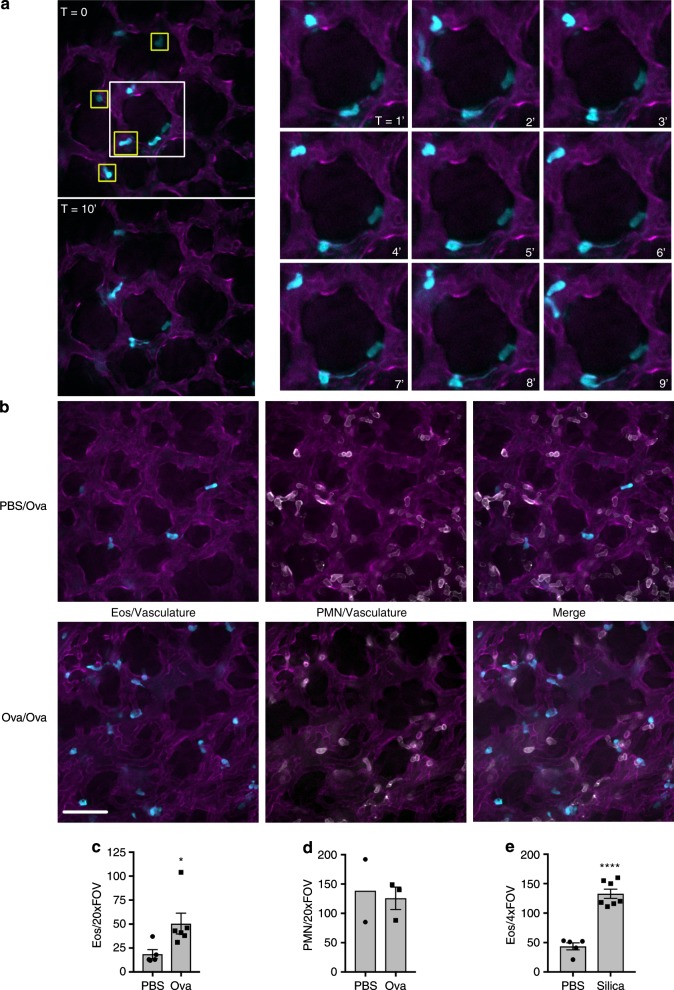
Intravital imaging of the lung under homeostatic conditions and following ovalbumin sensitization and challenge. **a** eoCRE^+/−^/tdTomato^+/−^ mice were anesthetized and prepared for lung intravital microscopy without any stimulation as described in the “Methods.” Eosinophils express tdTomato (pseudo-colored cyan for consistency) and the vasculature is labeled with an anti-CD31 antibody conjugated to Alexa-488 (pseudo-colored magenta for consistency). Representative images were captured from video sequences to show eosinophils in the lung under baseline conditions that either interacted transiently with the vasculature (yellow boxes) or that patrolled the lung (time series on the right). Patrolling cells were intravascular as demonstrated by dual labeling with anti-CD45 (Supplementary Fig. 3). **b**–**d** eoCRE^+/−^/tdTomato^+/−^ mice were sensitized with either phosphate-buffered saline (PBS) or ovalbumin (Ova) and then challenged with aerosolized Ova as described in the “Methods.” Forty-eight hours after the last Ova challenge, mice were anesthetized and prepared for intravital microscopy. Eosinophils express tdTomato (cyan), polymorphonuclear (PMN) cells were labeled with anti-Ly6G antibody conjugated to Alexa-647 (white), and the vasculature is labeled with an anti-CD31 antibody conjugated to Alexa-488 (magenta). Representative images captured from video sequences are shown in **b** and the quantified data for eosinophils and neutrophils (PMN) are shown in **c**, **d**, respectively. Data for eosinophils represent the mean ± SEM for *n* = 5 independent experiments (PBS) or *n* = 6 independent experiments (Ova). Data for neutrophils represent the mean and range of two experiments for PBS and mean ± SEM for *n* = 3 experiments for Ova. *p = 0.0341. **e** eoCRE^+/−^/tdTomato^+/−^ mice were anesthetized and saline alone or saline with silica particles was instilled intranasally as described in the “Methods.” After 12 h, animals were anesthetized and prepared for intravital microscopy. Video sequences were captured and the number of eosinophils/×4 field of view was determined. The data shown in **e** represent mean ± SEM for *n* = 5 independent experiments (PBS) or *n* = 7 independent experiments (Silica). ****p < 0.0001. Scale bars are 50 μm

We used the method first described by Thornton et al.^32^ and modified by Yipp et al.^33^ to visualize eosinophils in the lung under baseline conditions using intravital microscopy. This method combined with spinning-disk confocal microscopy enables imaging of vessels present at depths of up to 25 μm from the surface of the exposed lung. Lung capillaries were labeled with fluorescently conjugated anti-CD31 (magenta), leaving the alveolar spaces unlabeled (black). We found that a few eosinophils were consistently present in the lung vasculature under baseline conditions (Fig. 4a), which was consistent with previously published reports^21^ and our whole-body imaging (Fig. 3). Eosinophil retention in the lung was typically transient, with cells rapidly detaching and re-entering the blood flow (Fig. 4a, yellow boxes).

We also observed eosinophils patrolling the vasculature (Fig. 4a, time series). In some cases, these cells had long, mobile processes and remained within the imaging field for the duration of the 10-min video recording (Fig. 4a and supplementary video 2). To determine whether these eosinophils were crawling within the blood vessels or whether they were extravascular, we injected animals intravenously with anti-CD45. CD45 is found on circulating leukocytes and intravenous injection with anti-CD45 is a method previously used to differentiate between those leukocytes that are within the vascular from those that have extravasated^34^. We found that these patrolling eosinophils also labeled with anti-CD45, suggesting that, at the time of injection, they were within the blood vessels (Supplementary Fig. 3).

We next examined eosinophil recruitment and behavior during airway inflammation using the well-characterized ovalbumin (Ova) sensitization and challenge model. Animals were sensitized with an intraperitoneal (IP) injection of either phosphate-buffered saline (PBS) or Ova on days 1 and 14 and then challenged with aerosolized Ova on days 24, 25, and 26^5^. The lungs were then examined on day 28^5^. When we examined the bronchoalveolar lavage in this model, there was a four-fold increase in total cellularity following Ova sensitization and challenge (PBS: 1.8 × 10^5^ ± 0.3 cells/mL and Ova: 7.9 × 10^5^ ± 2.4 cells/mL; *p* = 0.0023, *n* = 5) and the cellular infiltrate was predominately eosinophils (Ova 47.16 ± 7.4%, *n* = 5), as previously reported.

A few eosinophils were present in the lungs of PBS-injected mice (Fig. 4b, PBS/Ova), similar to what was seen in naive animals (Fig. 4a). Sensitization and challenge with Ova led to a significant increase in the total number of eosinophils in the lung (Fig. 4b, Ova/Ova and quantified in Fig. 4c). We also visualized neutrophils (white) in these experiments by labeling neutrophils with a fluorescently conjugated anti-Ly6G antibody in the eoCRE^+/−^/GFP^+/−^ or eoCRE^+/−^/tdTomato^+/−^ mice. As previously reported, neutrophils were constitutively present in the lung under baseline conditions (Fig. 4b)^33^. Ova sensitization and challenge had no effect on neutrophil recruitment at the time points examined (Fig. 4b, d). To determine whether eosinophil recruitment could be measured in another model of lung inflammation, we exposed mice to silica particles to induce acute injury. Eosinophil recruitment was then measured 12 h later. Silica-induced injury led to a three-fold increase in eosinophil recruitment 12 h following exposure (Fig. 4e).

**Fig. 5:**
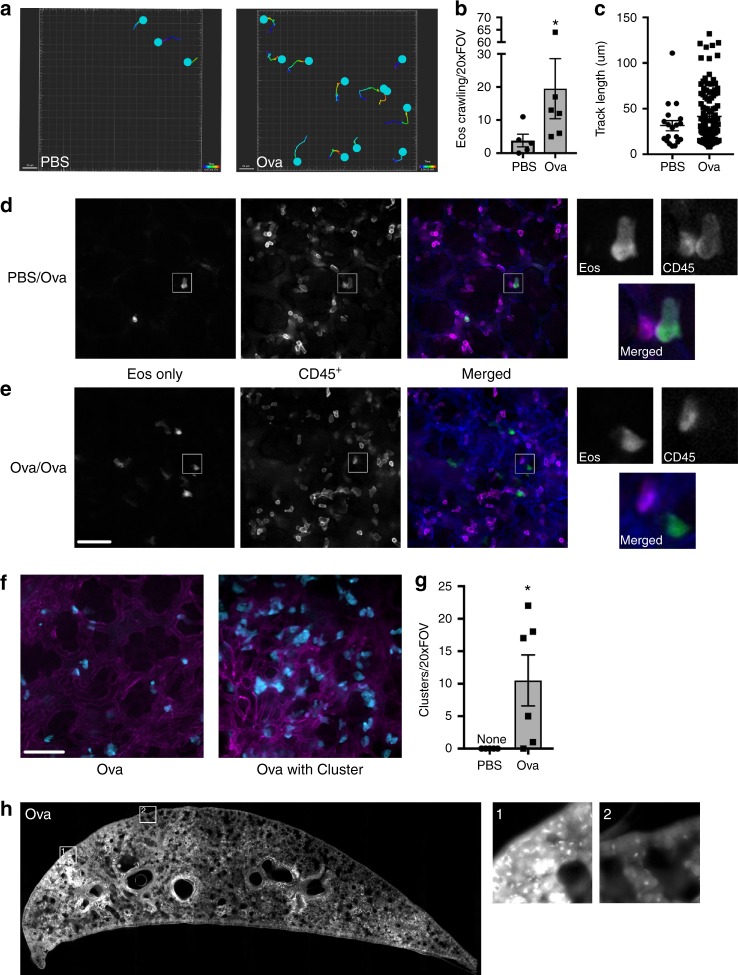
Eosinophil behavior and distribution in the lung following ovalbumin sensitization and challenge. EoCRE^+/−^/tdTomato^+/−^ mice were injected with either phosphate-buffered saline (PBS) or ovalbumin (Ova) and then challenged with aerosolized Ova as described in the “Methods.” Forty-eight hours after the last Ova challenge, mice were prepared for intravital microscopy of the lung. **a** The migration tracks of eosinophils from either PBS/Ova (PBS) or Ova/Ova (Ova) animals are shown. The number of crawling eosinophils (**b**) and the distance traveled (**c**) were measured. Data in **b** are mean ± SEM and individual values from *n* = 5 independent experiments (PBS) or *n* = 6 independent experiments (Ova) (*p = 0.0173) and the data in **c** show the individual measurements of track length. **d**, **e** Prior to intravital imaging of eoCRE^+/−^/tdTomato^+/−^ mice, animals were given an intravenous injection of anti-CD45 conjugated to Alexa-647. Images from individual channels are shown in grayscale. Merged images show eosinophils in green, intravascular leukocytes labeled with anti-CD45 antibody in magenta, and the vasculature in blue. Data represent three independent experiments. **f** Dispersed eosinophils or eosinophils in clusters are shown in **f** and the extent of clustering is quantified in **g**. Data in **g** represent the mean ± SEM of *n* = 5 independent experiments (PBS) or *n* = 6 independent experiments (Ova). *p = 0.0152. **h** Lungs from Ova/Ova eoCRE^+/−^/tdTomato^+/−^ mice were fixed and imaged as described in “Methods.” Scale bars are 50 μm

We went on to examine eosinophil behavior in this model. We found that eosinophils were highly motile following Ova sensitization and challenge. Figure 5a shows the migration tracks of eosinophils in either PBS or Ova-treated animals over a 10-min interval. When this behavior was quantified, significantly more eosinophils exhibited a crawling behavior in the Ova-treated animals as compared to those treated with PBS (Fig. 5b), and they generally migrated greater distances (Fig. 5c). Notably, we observed the occasional patrolling eosinophil in the PBS-treated animals as demonstrated by the outlier in Fig. 5c. To determine whether eosinophils were crawling within the blood vessels or whether they had exited the blood stream, we injected animals intravenously with anti-CD45 to differentiate between those leukocytes that are within the vascular from those that have extravasated^34^. In PBS-sensitized mice, eosinophils were only found within the vasculature, as demonstrated by anti-CD45 labeling of all tdTomato-expressing cells (Fig. 5d), resulting in eosinophils being dual labeled (Fig. 5d, inset images (a) Eos only, (b) CD45^+^, (c) Merged). In contrast, eosinophils in Ova-treated animals were both intravascular and extravascular (Fig. 5e, inset images (a) Eos only, (b) CD45^+^, (c) Merged).

Another notable feature in the Ova-treated mice was that eosinophils were often found in clusters (Fig. 5f, quantified in Fig. 5g). Examining the individual points from each experiment shows that cluster formation was bimodal, suggesting non-uniformity in cluster formation. Clusters were not evident in saline-treated animals, suggesting this was not a response induced by the surgical procedure. To determine whether this was a local phenomenon or whether these clusters were present throughout the lung, we initially examined whole lungs from PBS- and Ova-treated mice ex vivo using whole-body imaging; however, the resolution was limited. To further examine eosinophil distribution following Ova challenge and stimulation, we took advantage of the fact that tdTomato fluorescence is preserved following fixation^35^ and imaged a cross-section of the fixed lung. We found that eosinophils had a patchy distribution in Ova-treated animals with eosinophil clusters clearly evident (Fig. 5h, inset 1: clustered, inset 2 not clustered).

## Discussion

We developed a model to directly examine eosinophils in vivo enabling us to look more deeply into the behavior of this enigmatic cell. Eosinophils are found across all five classes of vertebrata and they can be found in most parts of the human body^1,2,23^, but the question remains—what are they doing? Our knowledge of how eosinophils traffic in humans has historically been limited to end-point histology. More recently, in vivo tracking of ^111^Indium- or Technetium-99m-labeled eosinophils in healthy patients or patients with inflammation has expanded our understanding of the kinetics of eosinophil trafficking in humans^36–40^, yet these studies are limited to low-resolution imaging of previously isolated and re-injected eosinophils. Further, the question of normal eosinophil behavior has been confounded by data showing that patients receiving anti-IL-5 therapy or the few individuals that are congenitally deficient in eosinophils seem to suffer no consequences due to the lack of eosinophils^41,42^. Animal models have been used to try and understand these findings, yet animal studies have been limited to knock out strategies and by models that rely on end points or surrogates^2,23,43^. The eoCRE mice have been a key tool in unraveling the behavior of these cells in mice, yet to date reporters using this model have only been used to confirm specificity or examine fixed tissue^12,27,44^. Combining intravital microscopy with these reporter mice not only allows us to count tissue eosinophils, but more importantly, it also provides a new tool for studying eosinophil behavior in health and disease.

We can now see that tissue eosinophils in a healthy animal are not resting. Intravital microscopy shows that they are spread-out in the cecum (Fig. 1e), crawling through the lymph node (Fig. 2g), degranulating in the colon (Fig. 1f) and skin (Fig. 2f), and a select few are patrolling the lung (Fig. 4a). This lends support to the concept of eosinophil subpopulations with specific roles dependent on location that has been put forward by several groups. For example, Mensil et al. identified an IL-5-independent resident population of eosinophils in the lung with a unique transcriptomic profile^21^.

Further, Arnold et al. showed that under normal conditions eosinophils in the GI tract actively suppress Th1 responses in an interferon-γ-dependent manner^44^ and Xenakis et al. found two subsets of eosinophils in the intestinal lamina propia^12^. Notably, Xenakis et al. used a similar strategy to generate fluorescent eosinophils in situ and then examined them within the villi of the small intestine in fixed tissue sections^12^. Our data in the GI tract also show evidence for differential activation of eosinophils. We examined the muscular layer as opposed to the submucosa and found that eosinophils in this region of the small intestine were rounded, whereas those in the cecum and colon were spread with numerous processes consistent with a more activated state (Fig. 1). C-C chemokine motif ligand 11 is important for baseline homing of eosinophils to the gut^16,45^, yet there must be other factors at work that lead to these differences in morphology. Compartment-specific chemokines, changes in tissue architecture or microbiota for example^46^, could influence eosinophil activation, which can now be tested using these reporter mice.

Eosinophils have the machinery and can act as antigen-presenting cells^46–49^. They traffic to lymph nodes following activation and interact with T cells to influence their differentiation^20^. We visualized eosinophils in the healthy lymph node and found that they displayed a wide scope of movement and morphology. Their behaviors ranged from being highly motile to nearly stationary and from rounded to flattened (Fig. 2c, d, g), which suggests that each subset may be fulfilling a different function. Simultaneous multi-cellular imaging has revealed much about the behavior of other immunocytes in the lymph node. We can now include eosinophils and determine how they communicate with others cells within this rich immune environment.

A striking find was observing small punctate fluorescent structures consistent with intact granules in healthy tissue. This was unexpected outside of the GI tract^22^ because the release of intact granules is thought to only occur during eosinophil cytolysis, generally considered a violent pro-inflammatory process^28,50^. If these are confirmed to be crystalline core containing granules, this raises several intriguing questions. Are these granules still being released via cytolysis? What purpose do they serve in healthy tissues? Decorated with receptors for eotaxins, leukotrienes, and cytokines, could they be landmines poised to respond to inflammatory challenges? With the reporter mice described here, we can now begin to answer these new questions.

There is a wealth of data on eosinophils in the lung, yet visualizing these cells in vivo has remained elusive. Combining lung intravital microscopy with eoCRE reporter mice revealed a population of eosinophils that patrol the lung under normal conditions (Fig. 4a). In some cases, these resident cells had long processes previously observed in eosinophils residing in the intestinal tract^12^. Future studies will determine whether these are the same resident population of eosinophils described by Mesnil et al.^21^. Eosinophil numbers increased following Ova sensitization and challenge as expected (Fig. 4b, c). By directly visualizing eosinophils, we were also able to measure behaviors including crawling, migration and clustering (Fig. 5), each of which may be regulated differently. We were also able to differentiate between intravascular and extravascular eosinophils (Fig. 5b), which will enable the process of eosinophil extravasation in the lung to be thoroughly and directly examined. Additionally, by imaging eosinophils with neutrophils in the Ova-challenged lung, we showed differential activation of eosinophils while neutrophils remained unaffected (Fig. 4b–d). Going forward, we can investigate eosinophil interactions with other cells including B cells, T cell subsets, and macrophages during allergic inflammation.

The list of tissues and conditions where eosinophils are present are many, yet our answers about what they are doing are still few. Now we have a tool to further delve into the question of what tissue-resident eosinophils are doing under steady-state conditions and whether there are eosinophil subpopulations. Eosinophil-specific fluorescent reporter mice will enable us to answer more of these questions. The tdTomato reporter mouse is particularly useful due to the stability of the fluorescent marker, enabling not only intravital microscopy but also efficient whole-body imaging and histologic detection without needing to rely on stains or antibodies. These eosinophil reporter mice are a novel tool that will aid in learning more about eosinophil function in health and disease.

## Methods

### Animals

Animal experiments were conducted in accordance with the Canadian Council for Animal Care guidelines and following approval from the University of Calgary Animal Care Committee or in accordance with National Institutes of Health and Mayo Foundation institutional guidelines. Six-to-8-week-old C57Bl/6, B6.Cg-Gt(ROSA)26Sor^tm6(CAG-ZsGreen1)Hze^/J, and B6.Cg-Gt(ROSA)26Sor^tm9(CAG-tdTomato)Hze^/J mice were purchased from Jackson Laboratories (Bar Harbor, ME, US). EoCRE mice were previously described^27^ and generously provided by Dr. J.J. Lee at the Mayo Clinic, Scottsdale. A key finding was that heterozygote mice expressed sufficient amounts of CRE recombinase to enable excision of targets expressed at the flox-stop-flox locus in ROSA26 while maintaining sufficient amounts of EPX^27^. EoCRE^+/−^/eGFP^+/−^ and eoCRE^+/−^/tdTomato^+/−^ mice colonies were bred, housed, and maintained at the University of Calgary Animal Resource Centre. All imaging experiments were carried out at the Live Cell Imaging Laboratory at the University of Calgary.

### Antibodies

The ant-eosinophil peroxidase (MM25.82.2) was sourced from the J.J. Lee laboratory and validated in ref. ^36^. All other antibodies were commercially sourced and validated. These antibodies include: anti-CD31 conjugated to Alexa 488 (Clone 390, BioLegend, Catalog #102414, lots B195379, B228833, B239624), anti-CD31 conjugated to Alexa 647 (Clone 390, BioLegend, Catalog #102416, lots B197826), anti-CD31 conjugated to Alexa 594 was conjugated in house using anti-CD31 (Clone 390, BD Biosciences, Catalog #553708, lot 5324862) and Alexa Fluor 594 Protein labeling kit (Life Technologies, Inc, Catalog #A10239, lot 1724776), anti-Ly6G conjugated to Alexa 647 (Clone 1A8, BioLegend, Catalog #127610, lots B204928, B255839), and anti-CD45.2 conjugated to Alexa 647 (Clone 104, BioLegend, Catalog #109818, lot B194090).

### Histology for EPX

Tissues were isolated from 11-week-old, female C57Bl/6 mice, fixed in 10% formalin for 24 h at 4 °C, paraffin embedded, and sliced into 5-µm sections. Sections of tissues were stained as in our earlier publications using both immunohistochemistry with a rat monoclonal antibody specific for MBP-1^51^ or a mouse monoclonal antibody specific for EPX (MM25.82.2)^43^. All DAKO reagents were from DAKO Agilent Technologies, Inc. (Santa Clara, CA, USA). Briefly, slides were deparaffinized, rehydrated, and underwent antigen retrieval with DAKO Target Retrieval solution. Slides were then treated with DAKO Dual Endogenous Blocking solution for 10 min, followed by Pepsin-Digest All 3 (Invitrogen, Carslbad, CA, USA) for 10 min., Slides were then blocked with 2.5% donkey serum or rodent block M (Biocare Medical, Pacheco, CA, USA) for 30 min. Between each blocking/treatment step, slides were washed three times with DAKO Wash solution. EPX antibody was added at a 1:300 dilution in DAKO diluent with background-reducing component and incubated overnight at 4 °C. After washing, biotinylated goat anti-mouse IgG antibody (Vector Laboratories, Dossenheim, Germany) was added at a 1:500 dilution and incubated at room temperature for 30–45 min. After washing, slides were treated with ABC reagent (VECTASTAIN® ABC-AP Staining Kit, Alkaline Phosphatase, Standard from Vector Laboratories, Dossenheim, Germany) for 30 min, washed, processed with DAKP chromagen Permanent Red Substrate System, washed, and then counterstained with DAKO methyl green for 1 min. Slides were then washed, dried, and mounted with ClearMount (American Master Tech Scientific Lodi, CA). Tissue sections were imaged using a ×20/0.75 NA objective and digitized using a digital pathology scanner (Aperio AT Turbo, Leica Biosystems, Buffalo Grove, IL). The total area of each tissue section was calculated along with the number of eosinophils per section. The sum and standard error of counts for three sections (one per mouse) is calculated using the Aperio ImageScope software (version 11.2.0.780, Aperio Technologies, Vista, CA). Digitized images were extracted from Aperio at a magnification of ×200 for representative display in this manuscript.

### Intravital microscopy

Mice, 10–12-week old, were anesthetized by IP injection of 150 mg/kg of ketamine and 10 mg/kg of xylazine. The jugular vein was subsequently cannulated to allow for intravenous administration of additional ketamine and xylazine as well as fluorophore-conjugated antibodies. Five to 2.5 µg per mouse of the appropriate fluorophore-conjugated antibodies were used for intravital lung imaging. The antibodies used were mouse anti-CD31 (Clone 390), mouse anti-Ly6G (Clone 1A8), and mouse anti-CD45.2 (Clone 104), all from BioLegend (San Diego, CA, USA). Anti-CD31 was used to label the vasculature. Leukocytes, including eosinophils, are also CD31 positive; however, with the exposure setting used in this study, CD31 expression on leukocytes is too low to be visualized as compared to the bright vasculature. Anti-Ly6G was used to label neutrophils, which are Ly6G^hi^. Ly6G has been reported on subsets of eosinophils^52^. When neutrophils were quantified, cells that were double positive for Ly6G and tdTomato were excluded. Body temperatures of mice were monitored with a rectal probe attached to a Physitemp TCAT-2LV Controller. Mice were kept on a heated plate set to 38 °C and also warmed with a custom-built far infrared heating enclosure.

Intravital imaging for the GI tract, liver, skin, and lymph node were done as previously described^53–57^ and reviewed in ref. ^58^. Intravital microscopy of the small intestine specifically involved imaging through a coverslip in the middle of the small intestine, in the region of the jejunum. Imaging was performed by starting at the outside of the intestine and focusing into the muscular layer of the intestine, about 30–50 μm below the intestinal surface. Intravital imaging of the lung was performed as described^32,33^. Briefly, a tracheotomy was performed followed by the insertion of ventilation tubing with an inner diameter of 0.8 mm and an outer diameter of 1.2 mm. Mice were ventilated at 150 breaths per minute, a stroke volume of 150 µL, and a positive-end expiratory pressure of 3 cm H_2_O. Mice were placed on their right side, the thoracic cavity exposed, and a cover-slipped thoracic suction window was attached to the lung using a vacuum pressure of 50 mm Hg. Mice were imaged using either a × 10/0.3 NA objective or a ×20/0.95 NA objective with either a Quorum Wave FX-X1 spinning disk microscope or a Nikon A1R confocal (liver only). Images were captured and analyzed using the Volocity 6.3.1 Software (PerkinElmer, USA), FIJI (ImageJ, version 2.0.0-rc-68/1.52e)^59^, or Imaris software (versions 9.1.2 and 9.2.1, Bitplane, Oxford Instruments, Concord, MA, USA).

To quantify cell number, circularity, and area, FIJI (ImageJ) was used. Grayscale image stacks were converted to 8-bit and adjusted for brightness and contrast. An automatic triangle thresholding routine was applied to all frames to segment the eosinophils from the background. The analyze particle function was then applied to the resulting binary image and the total number of eosinophils, their total area, and the lengths of the major and minor axes of each eosinophil was determined. Total eosinophils are reported as cells/mm^2^, area (spreading) is reported as μm^2^, and circularity is unit-less. For data presentation, images were pseudocolored in FIJI and brightness and contrast changes were applied to the entire image. Files were then imported into Adobe Photoshop CS6.0, converted to 300 dpi, and blackpoint normalized. All final images were compared to the original raw image to ensure data integrity.

### Whole-organ imaging

To screen whole organs ex vivo, the organs from wild-type, eoCRE^+/−^/GFP^+/−^, or eoCRE^+/−^/tdTomato^+/−^ mice were dissected and then visualized using an InVivo Xtreme 4MP whole-body imaging system (Bruker, Billerica, MA). Green and red channels were visualized using 470 nm excitation and 535 nm emission or 540 nm/600 nm wavelengths, respectively. The imaging protocol contained two steps: reflectance imaging (2 s exposure time) and fluorescent imaging at the given wavelengths (5 s exposure time). Images from the InVivo Xtreme were acquired and analyzed using the Bruker molecular imaging software MI SE (version 7.1.3.20550). The fluorescent intensity was quantified by measuring the mean fluorescence signal intensity (corrected by background) in a constant region of interest for each individual organ.

### Inducing lung inflammation

Mice were sensitized to chicken Ova from Sigma-Aldrich (Oakville, ON, Canada) using the protocol reported by Jacobsen et al.^5^ with the modifications described here. Briefly, Ova was prepared at 0.909 mg/mL in PBS and mixed as per the manufacturer’s instructions with Imject Alum (Thermo Scientific, Waltham, MA, USA) at a ratio of 11:14 of Ova to Imject Alum. Mice were first sensitized to Ova by IP injection of 100 µL of either PBS or Ova/Imject Alum mix on days 0 and 14. On days 24, 25, and 26, mice were challenged with a 25-min exposure to nebulized 1 mg/mL Ova in saline. For the challenge, mice were housed in a pie cage (Braintree Scientific, Braintree, MA, USA) that received nebulized Ova from a Schuco cup nebulizer, with pressure on the vacuum pump set at <1 kg/cm^2^. Mice were imaged on day 28.

Acute silica exposure was performed similar to Ferreira et al.^60^. Briefly, mice were anesthetized with isofluorane and then instilled intranasally with sterile saline alone or sterile saline containing 10 mg crystalline silica with a particle size of 0.5–10 μm (Sigma Chemical, St Louis, MO). Twelve hours later, animals were prepared for lung intravital microscopy as described above.

### Statistics and reproducibility

All experiments were performed between three and five times unless otherwise specified. Results are expressed as a mean ± SEM and the individual values are shown. An unpaired two-tailed Student’s *t* test was used to compare two groups with normal data distribution and Mann–Whitney was used to compare two groups without a normal distribution. One-way analysis of variance was performed to analyze differences in the mean values across more than two groups. This was followed by the appropriate post-test to assess differences between groups. *p* Values <0.05 were considered to be statistically significant.

Animals were allocated into particular groups based on sex and age. Beyond that, allocation was random. Blinding was not possible for most of this work. For data acquisition, the researcher performing the surgical and microscopy work is also the person who collected the data from the living animal while monitoring and maintaining it on the microscope. To aid in generating unbiased data, fields of view were selected based on imaging the vasculature first (CD31). Only after the field of view was chosen were the eosinophils visualized and the data collected. For analysis, the morphology and vasculature of the organ were immediately evident. Analysis was automated and standardized to minimize bias in counting cells. All attempts to reproduce the data were successful.

### Reporting summary

Further information on research design is available in the Nature Research Reporting Summary linked to this article.

## Supplementary information

Supplemental Information

Description of Additional Supplementary Files

Reporting Summary

Supplemental Video 1

Supplemental Video 2

Supplementary Data

## Data Availability

Source data are available as Supplementary Data. All other data generated or analyzed during this study are available from the corresponding author on reasonable request.
