# Supplementary material for: Intravital imaging allows real-time characterization of tissue resident eosinophils
Source: Commun Biol. 2019 May 13;2:181. doi: 10.1038/s42003-019-0425-3 (PMC6513871; doi:10.1038/s42003-019-0425-3)
Supplement: Supplementary file 1 — Supplemental Information [file 42003_2019_425_MOESM1_ESM.pdf]

Supplementary Information

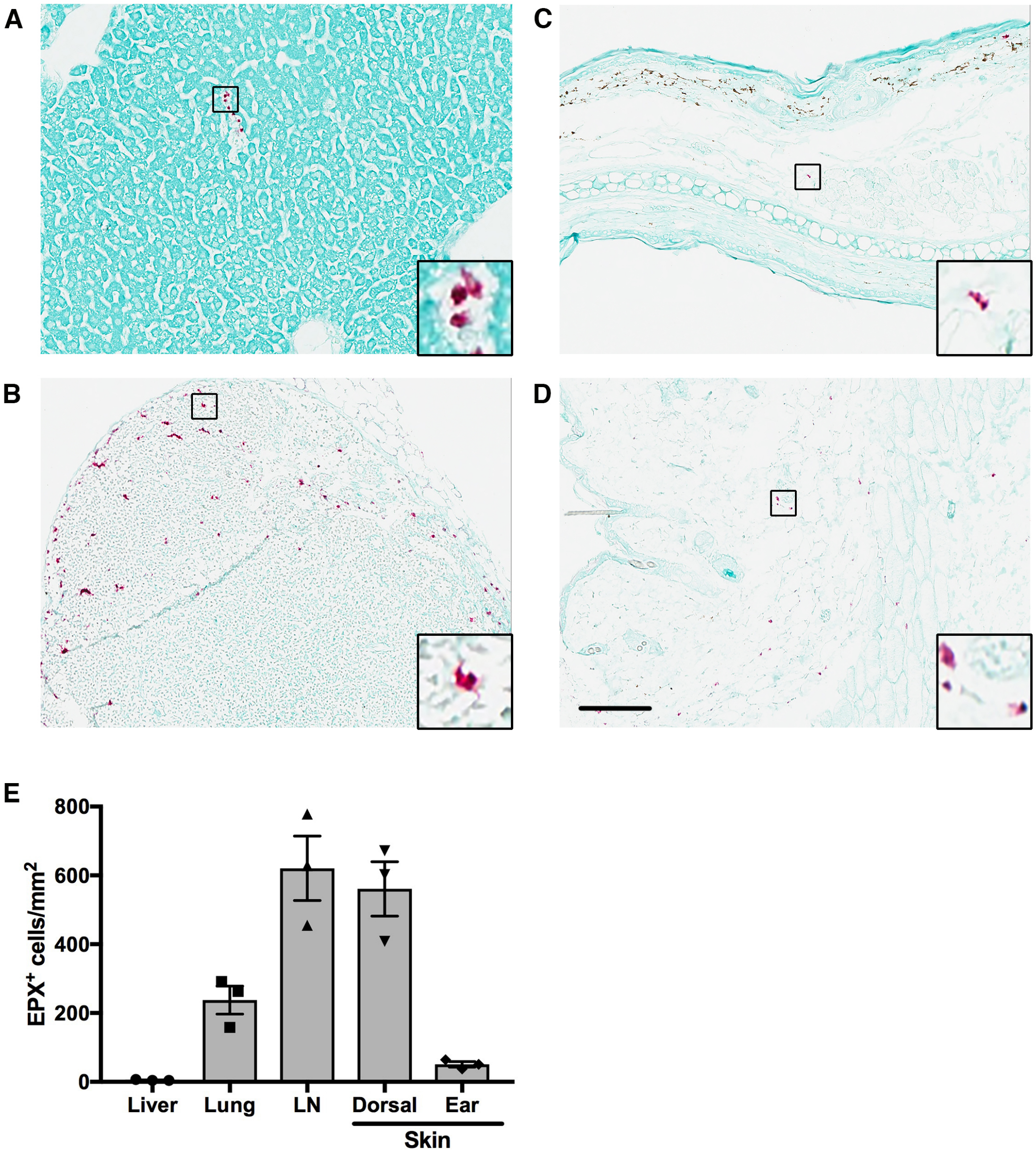

Supplementary Figure 1. Eosinophils are expressed in the liver, lymph node and skin under baseline conditions. (A-D) Tissues from wild-type C57Bl/6 mice were isolated, fixed, sectioned and then stained with an anti-eosinophil peroxidase (EPX) antibody as described in the *materials and methods*. Slices were imaged on a digital pathology slide scanner using a 20x/0.75NA objective. Representative images are shown in A-D. The digitized data were quantified and the data shown in E are the mean ± SEM and individual values of three independent experiments. **P<0.01 between the indicated groups. Scale bar for A-D is shown in D and is 100 μm.

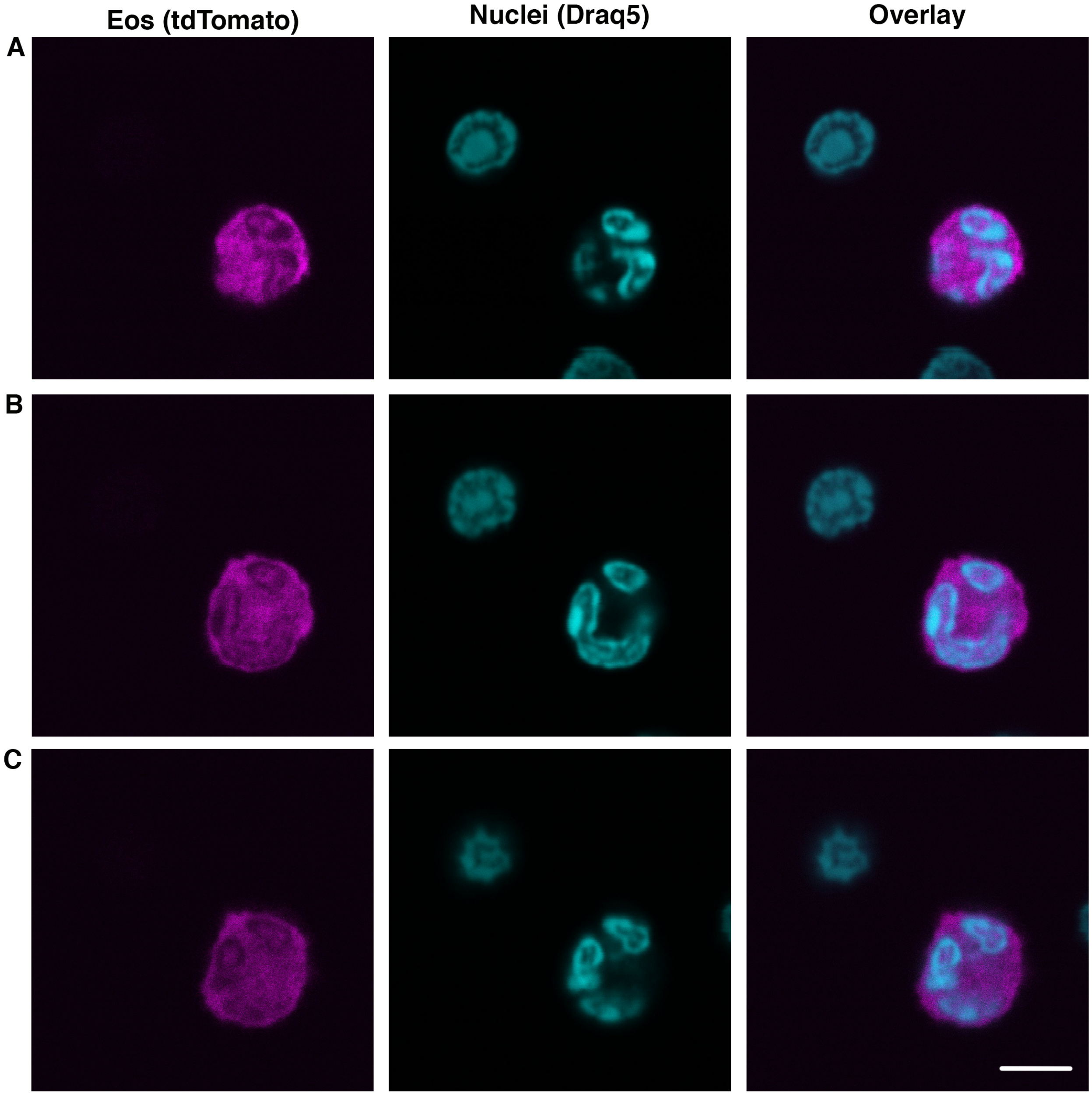

Supplementary Figure 2. Eosinophils from eoCRE$^{+/-}$/tdTomato$^{+/-}$ mice are uniformly fluorescent. Blood was harvested from three (3) naive eoCRE/tdTomato mice by cardiac puncture. The red cells were lysed and the leukocytes were imaged using laser scanning confocal microscopy (Nikon A1R) with a 60x/1.4 NA objective. 100 nm steps were taken to optically section the eosinophil. Eosinophils were identified by their fluorescence in the red channel (ex: 561nm, em: 570-620nm; shown as magenta). Draq5 (ex: 640, em: 663-738 nm; shown as cyan) was used to label the nuclei of all leukocytes. (A-C) Representative images from three separate optical sections are shown. Each section was 10 steps or 1 μm apart. Scale bar is 10 μm.

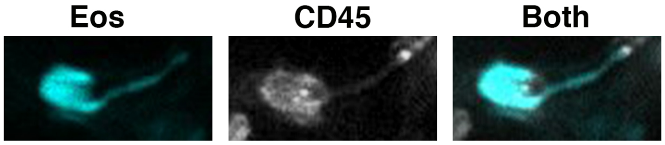

Supplementary Figure 3. Patrolling eosinophil in the lung is within the vasculature. eoCRE$^{+/-}$/tdTomato$^{+/-}$ mice were anesthetized and prepared for lung intravital microscopy without any stimulation as described in the *materials and methods*. Eosinophils expressing tdTomato are pseudo-colored cyan for consistency. Animals were given an iv injection of anti-CD45 conjugated to Alexa-647 as described in figure 5. Images from individual channels are shown with Eos in cyan and CD45 in greyscale or both together. This cell is the same as shown in figure 4A at nine minutes.
