# Supplementary material for: Intravital imaging allows real-time characterization of tissue resident eosinophils
Source: Commun Biol. 2019 May 13;2:181. doi: 10.1038/s42003-019-0425-3 (PMC6513871; doi:10.1038/s42003-019-0425-3)
Supplement: Supplementary file 2 — Description of Additional Supplementary Files [file 42003_2019_425_MOESM2_ESM.pdf]

## Description of Additional Supplementary Files

**File Name**: Supplementary Movie 1

**Description**: **Eosinophils in the lymph node.** EoCRE+/- /GFP+/- mice were anesthetized and the inguinal lymph node was prepared for intravital microscopy as described in methods. Eosinophils expressing GFP (cyan) were imaged using video microscopy. Images were captured every 1.3 seconds for a total of 6 minutes. Video is shown at 23 frames per second, representing a 30x speed as compared to real time. Image frames spanning 90 seconds are shown in figure 2G.

**File Name**: Supplementary Movie 2

**Description**: **Eosinophils in the lungs of naïve mice**. EoCRE+/- /tdTomato+/- mice were anesthetized and the lung was prepared for intravital microscopy as described in methods. Eosinophils express tdTomato (pseudo-colored cyan for consistency) and the vasculature is labeled with an anti-CD31 antibody conjugated to Alexa-488 (pseudo-colored magenta for consistency). Images were captured every 5 seconds for a total of 10 minutes. Video is shown at 10 frames per second, representing a 50x speed as compared to real time. Image frames are shown in figure 4B.

**File Name**: Supplementary Data

**Description**: Source data for all graphs provided in Excel format.
