# Supplementary material for: Intravital imaging allows real-time characterization of tissue resident eosinophils
Source: Commun Biol. 2019 May 13;2:181. doi: 10.1038/s42003-019-0425-3 (PMC6513871; doi:10.1038/s42003-019-0425-3)
Supplement: Supplementary file 3 — Reporting Summary [file 42003_2019_425_MOESM3_ESM.pdf]

nature research

Corresponding author(s): Kamala D. Patel

Last updated by author(s): Mar 27, 2019

Reporting Summary

Nature Research wishes to improve the reproducibility of the work that we publish. This form provides structure for consistency and transparency in reporting. For further information on Nature Research policies, see Authors & Referees and the Editorial Policy Checklist.

Statistics

For all statistical analyses, confirm that the following items are present in the figure legend, table legend, main text, or Methods section.

n/a	Confirmed	
☐	☒	The exact sample size (*n*) for each experimental group/condition, given as a discrete number and unit of measurement
☐	☒	A statement on whether measurements were taken from distinct samples or whether the same sample was measured repeatedly
☐	☒	The statistical test(s) used AND whether they are one- or two-sided<br>*Only common tests should be described solely by name; describe more complex techniques in the Methods section.*
☒	☐	A description of all covariates tested
☐	☒	A description of any assumptions or corrections, such as tests of normality and adjustment for multiple comparisons
☐	☒	A full description of the statistical parameters including central tendency (e.g. means) or other basic estimates (e.g. regression coefficient) AND variation (e.g. standard deviation) or associated estimates of uncertainty (e.g. confidence intervals)
☐	☒	For null hypothesis testing, the test statistic (e.g. *F*, *t*, *r*) with confidence intervals, effect sizes, degrees of freedom and *P* value noted<br>*Give P values as exact values whenever suitable.*
☒	☐	For Bayesian analysis, information on the choice of priors and Markov chain Monte Carlo settings
☒	☐	For hierarchical and complex designs, identification of the appropriate level for tests and full reporting of outcomes
☒	☐	Estimates of effect sizes (e.g. Cohen's *d*, Pearson's *r*), indicating how they were calculated

Our web collection on statistics for biologists contains articles on many of the points above.

Software and code

Policy information about availability of computer code

Data collection	Aperio ImageScope (version 11.2.0.780, Aperio Technologies, Vista, CA)<br>Volocity (version 6.3.1, PerkinElmer, US)<br>Bruker molecular imaging software MI SE (version 7.1.3.20550)
Data analysis	Aperio ImageScope (version 11.2.0.780, Aperio Technologies, Vista, CA)<br>Volocity (version 6.3.1, PerkinElmer, US)<br>Bruker molecular imaging software MI SE (version 7.1.3.20550)<br>Fiji/ImageJ (version 2.0.0-rc-68/1.52 e, NIH Open Source)<br>Imaris (version 9.1.2 and 9.2.1, Bitplane, Oxford Instruments, Concord, MA, US)

For manuscripts utilizing custom algorithms or software that are central to the research but not yet described in published literature, software must be made available to editors/reviewers. We strongly encourage code deposition in a community repository (e.g. GitHub). See the Nature Research guidelines for submitting code & software for further information.

Data

Policy information about availability of data

All manuscripts must include a data availability statement. This statement should provide the following information, where applicable:

- Accession codes, unique identifiers, or web links for publicly available datasets
- A list of figures that have associated raw data
- A description of any restrictions on data availability

The datasets generated during and/or analysed during the current study are available from the corresponding author on reasonable request.

Field-specific reporting

Please select the one below that is the best fit for your research. If you are not sure, read the appropriate sections before making your selection.

☒ Life sciences ☐ Behavioural & social sciences ☐ Ecological, evolutionary & environmental sciences

For a reference copy of the document with all sections, see nature.com/documents/nr-reporting-summary-flat.pdf

Life sciences study design

All studies must disclose on these points even when the disclosure is negative.

Sample size	In figures 1-3, the goal was to determine the number of eosinophils present under baseline conditions. These data were not being compared. In these cases a minimum of three different animals were used. A standard power calculation was performed to compare the number of eosinophils in PBS vs Ova. As a result we used a minimum of 5 animals. This is consistent with previously published work from our team.
Data exclusions	The only data excluded from this study was from a case in which the animal died before the end of the experiment. Unexpected death while under anesthesia is a pre-established criterion for data exclusion.
Replication	All attempts to reproduce the data were successful.
Randomization	Animals were allocated into particular groups based on sex and age. Beyond that, allocation was random.
Blinding	Blinding was not possible for most of this work. For data acquisition, the researcher performing the surgical and microscopy work is also the person who collects the data from the living animal while monitoring and maintaining it on the microscope. To aid in generating unbiased data, fields of view are selected based on imaging the vasculature first (CD31). Only after the field of view is chosen are the eosinophils visualized and the data collected. For analysis, the morphology and vasculature of the organ are immediately evident. Analysis was automated and standardized to minimize bias in counting cells.

Reporting for specific materials, systems and methods

We require information from authors about some types of materials, experimental systems and methods used in many studies. Here, indicate whether each material, system or method listed is relevant to your study. If you are not sure if a list item applies to your research, read the appropriate section before selecting a response.

Materials & experimental systems

n/a	Involved in the study	
☐	☒	Antibodies
☒	☐	Eukaryotic cell lines
☒	☐	Palaeontology
☐	☒	Animals and other organisms
☒	☐	Human research participants
☒	☐	Clinical data

Methods

n/a	Involved in the study	
☒	☐	ChIP-seq
☒	☐	Flow cytometry
☒	☐	MRI-based neuroimaging

Antibodies

Antibodies used	Eosinophil peroxidase (MM25.82.2, source JJ Lee laboratory)<br>anti-CD31 conjugated to Alexa 488 (Clone 390, BioLegend, Catalog #102414, lots B195379, B228833, B239624)<br>anti-CD31 conjugated to Alexa 647 (Clone 390, BioLegend, Catalog #102416, lots B197826)<br>anti-CD31 conjugated to Alexa 594 was conjugated in house using anti-CD31 (Clone 390, BD Biosciences, Catalog # 553708, lot 5324862) and Alexa Fluor 594 Protein labeling kit (Life Technologies, Inc, Catalog #A10239, lot 1724776)<br>anti-Ly6G conjugated to Alexa 647 (Clone 1A8, BioLegend, Catalog #127610, lots B204928, B255839)<br>anti-CD45.2 conjugated to Alexa 647 (Clone 104, BioLegend, Catalog #109818, lot B194090)
Validation	EPX antibody reference 36<br>anti-CD31 (Clone 390, BioLegend, Catalog #102414 and 102416), https://www.biolegend.com/en-us/products/purified-anti-mouse-cd31-antibody-123<br>Anti-CD31 (Clone 390, BD Biosciences, Catalog #553708)<br>http://www.bdbiosciences.com/us/applications/research/stem-cell-research/cancer-research/human/purified-nale-rat-anti-mouse-cd31-390/p/553708<br>mouse anti-Ly6G conjugated to Alexa 647 (Clone 1A8, BioLegend, Catalog #127610), https://www.biolegend.com/en-us/products/alexa-fluor-647-anti-mouse-ly-6g-antibody-4780<br>mouse anti-CD45.2 conjugated to Alexa 647 (Clone 104, BioLegend, Catalog #109818), https://www.biolegend.com/en-us/products/alexa-fluor-647-anti-mouse-cd45-2-antibody-3107

Animals and other organisms

Policy information about studies involving animals; ARRIVE guidelines recommended for reporting animal research

Laboratory animals	Species: Mice. Strain: C57Bl6. Sex and age: males and females aged 10-12 weeks. Specific strains included: C57Bl/6, B6.Cg-Gt(ROSA)26Sortm6(CAG-ZsGreen1)Hze/J and B6.Cg-Gt(ROSA)26Sortm9(CAG-tdTomato)Hze/J from Jackson labs and eoCRE from the JJ Lee laboratory.
Wild animals	The study did not involve wild animals.
Field-collected samples	The study did not involve samples collected from the field
Ethics oversight	Animal experiments were conducted in accordance with the Canadian Council for Animal Care guidelines and following approval from the University of Calgary Animal Care Committee or in accordance with National Institutes of Health and Mayo Foundation institutional guidelines.

Note that full information on the approval of the study protocol must also be provided in the manuscript.
